# Dental management considerations in a child with sequelae of infantile cortical hyperostosis (Caffey disease): a case report

**DOI:** 10.3389/fdmed.2026.1858800

**Published:** 2026-06-17

**Authors:** Aishani Baksi, Nishi Joshi, Amarshree A. Shetty, Kavita Rai, Swagata Saha, Manju Raman Nair

**Affiliations:** 1 Department of Paediatric and Preventive Dentistry, A B Shetty Memorial Institute of Dental Sciences, NITTE (Deemed to be University), Deralakatte, Mangaluru, Karnataka, India; 2 Department of Pedodontics and Preventive Dentistry, Dr. D Y Patil Dental College & Hospital, Dr. D Y Patil Vidyapeeth (Deemed to be University), Pune, Maharashtra, India

**Keywords:** Caffey disease, early childhood caries, general anesthesia, infantile cortical hyperostosis, mandibular hyperostosis, pediatric dental rehabilitation

## Abstract

**Aim and background:**

Infantile cortical hyperostosis (Caffey disease) is a rare inflammatory skeletal disorder characterized by episodic subperiosteal bone formation, commonly involving the mandible during early craniofacial development. Residual mandibular enlargement, feeding difficulty, and developmental challenges resulting from the disease may predispose affected children to early childhood caries (ECC). However, evidence guiding dental management in such patients remains limited. This report describes the dental management of a toddler with sequelae of infantile cortical hyperostosis, highlighting condition-specific considerations influencing treatment planning.

**Case description:**

A 2-year-10-month-old male with a history of infantile cortical hyperostosis, residual mandibular cortical thickening, gross motor developmental delay, and moderate acute malnutrition presented with severe ECC involving multiple cavitated dentinal lesions. Caries burden was assessed clinically using dmft criteria. Radiographic evaluation demonstrated diffuse mandibular cortical thickening without evidence of odontogenic infection. Considering high caries risk, extent of disease, limited cooperation, and systemic status, full-mouth rehabilitation under general anesthesia was undertaken. Treatment included glass ionomer restorations in posterior teeth, anterior composite restoration, extraction of a non-restorable molar, pit and fissure sealants, and topical fluoride application. Multidisciplinary preoperative assessment included evaluation of airway status and associated systemic considerations. The procedure was completed uneventfully, with satisfactory immediate postoperative recovery and short-term improvement in feeding.

**Conclusion:**

This case highlights the need for individualized, multidisciplinary dental management in children with sequelae of infantile cortical hyperostosis. While full-mouth rehabilitation under general anesthesia was successfully performed in this instance, findings from a single case should be interpreted cautiously. Further evidence is required to establish condition-specific dental management protocols.

## 1 Introduction

Infantile cortical hyperostosis (Caffey disease) is a rare, self-limiting inflammatory disorder of early infancy characterized by subperiosteal new bone formation and a clinical triad of fever, soft tissue swelling, and hyperirritability, often accompanied by episodic inflammatory exacerbations (1). The mandible is one of the most frequently affected bones, and craniofacial involvement may result in facial asymmetry, tenderness, and functional disturbances during a critical stage of craniofacial growth and primary dentition development (2). Although the disease generally resolves spontaneously, residual craniofacial changes and functional sequelae may interfere with feeding, oral function, and caregiverassisted hygiene practices, potentially affecting oral health (3). However, these associations remain insufficiently explored in the literature.

Early childhood caries (ECC) is among the most prevalent chronic diseases of childhood and is characterized by rapid progression, particularly in children exposed to biological, behavioral, or systemic risk factors (4). Young children with developmental delay, nutritional compromise, or chronic inflammatory conditions represent a particularly vulnerable group. Recurrent episodes of pain, feeding difficulty, altered dietary patterns, and caregiver reprioritization during medical illness may indirectly increase caries susceptibility (5). While these risk pathways are biologically plausible, there is a lack of disease-specific evidence linking infantile cortical hyperostosis to ECC, and current understanding is largely extrapolated from broader pediatric and medically compromised populations.

The management of extensive ECC in medically complex young children presents unique clinical challenges. Limited cooperation, high treatment need, and systemic comorbidities often necessitate comprehensive dental rehabilitation under general anesthesia to ensure safe and definitive care (6). Nevertheless, existing literature primarily addresses general indications for such management, with minimal discussion of condition-specific modifications required in children with sequelae of rare inflammatory skeletal disorders such as infantile cortical hyperostosis. To date, there is a paucity of published reports describing the oral health presentation and dental management of children with infantile cortical hyperostosis, resulting in a limited evidence base to guide clinical decision-making in this population.

This case report describes the comprehensive oral rehabilitation of a 2-year-10-month-old child with sequelae of infantile cortical hyperostosis, developmental delay, and moderate acute malnutrition presenting with extensive ECC. The aim is to highlight condition-specific considerations influencing dental treatment planning, perioperative assessment, and preventive care, rather than to establish generalized clinical recommendations.

## 2 Case presentation

A 2-year-10-month-old male child was referred to the Department of Pediatric and Preventive Dentistry for management of extensive early childhood caries requiring comprehensive rehabilitation. The child had a history of infantile cortical hyperostosis (Caffey disease) with residual mandibular enlargement, associated with gross motor developmental delay and moderate acute malnutrition and was under regular pediatric follow-up. Based on medical history, nutritional compromise, and developmental status, the child was considered medically and behaviorally high-risk for oral disease progression.

The child was born at 8½ months of gestation via elective lower-segment cesarean section (previous cesarean indication) with a birth weight of 1.4 kg. He required neonatal intensive care admission for 15 days due to low birth weight and was initially fed expressed breast milk via orogastric tube before transitioning to direct breastfeeding. No antenatal infections or perinatal complications were reported. Immunizations were up to date as per the national schedule. History of prematurity and low birth weight were considered additional biological risk factors contributing to increased susceptibility to early childhood caries.

At approximately 6 months of age, the child developed insidious-onset swelling over the midline of the mandible measuring approximately 5 × 5 cm, which progressively extended bilaterally toward the submandibular and preauricular regions. The episodes were associated with irritability, excessive drooling, feeding refusal, and intermittent high-grade fever lasting 2–3 days. The swelling partially regressed following antibiotic therapy but recurred intermittently, sometimes triggered by upper respiratory tract infections. Over time, persistent diffuse mandibular prominence remained as a residual sequela between episodes. These recurrent inflammatory episodes were considered to have potential indirect effects on feeding patterns, oral hygiene practices, and overall caries risk. Past medical history revealed that the child had previously undergone adenoidectomy with bilateral myringotomy and grommet insertion, which was uneventful.

**Figure 1:**
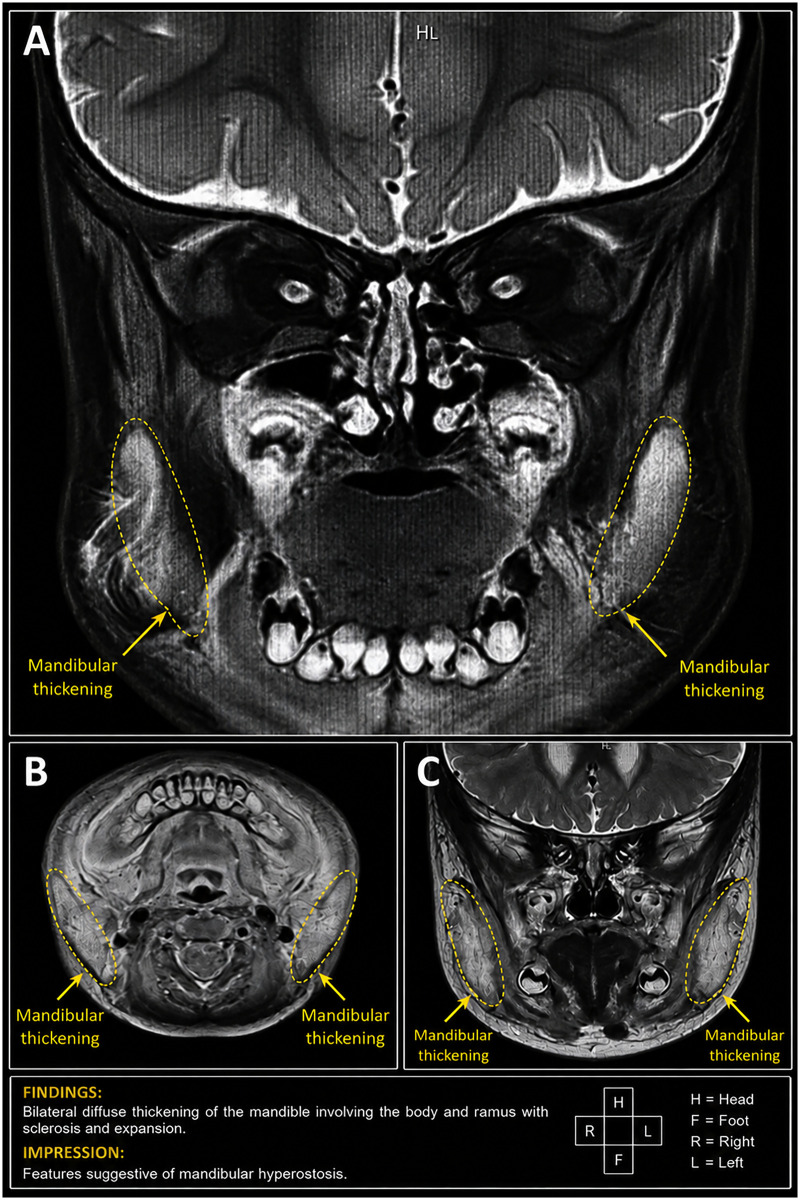
Multi-modal imaging assessment of a pediatric case of infantile cortical hyperostosis (Caffey disease) **(A)** coronal T2-weighted MRI demonstrating multiple unerupted tooth follicles within the maxillary and mandibular structures; **(B)** posterior coronal T2-weighted MRI highlighting diffuse soft tissue hyperintensity suggestive of masseteric myositis and developing permanent follicles; **(C)** axial T2-weighted MRI showcasing the overall dental arch form and relationship to unerupted maxillary dentition; three-dimensional computed tomography (CT) scan demonstrating diffuse cortical thickening of the entire mandible with pathognomonic periosteal reaction.

Extensive medical evaluation was undertaken. Fine-needle aspiration cytology demonstrated reactive lymphadenitis. Ultrasonography revealed bilateral submandibular and intraparotid lymphadenopathy. Magnetic resonance imaging showed increased masseter muscle thickness suggestive of myositis and multiple cervical and retropharyngeal lymph nodes. A three-dimensional computed tomography scan taken during infancy (at 6 months of age) demonstrated diffuse cortical thickening of the entire mandible with periosteal reaction and no lytic lesions, findings consistent with infantile cortical hyperostosis (Figure 1). Skeletal survey did not reveal significant involvement of other bones. Differential diagnoses including chronic osteomyelitis, metabolic bone disease, and infectious etiologies such as intrauterine TORCH infection were considered; however, clinicoradiological correlation supported the diagnosis of Caffey disease. The absence of lytic lesions, suppuration, or progressive bone destruction helped exclude chronic osteomyelitis and other infective pathologies. Neuroimaging also demonstrated periventricular white matter hyperintensities and calcifications with mild volume loss, the clinical significance of which was monitored by Department of Pediatrics.

Developmental assessment revealed significant delay in gross motor milestones, with head control achieved at approximately 1 year of age and independent walking attained at 2 years and 10 months (developmental quotient approximately 44% in the gross motor domain). Fine motor, language, and social milestones were comparatively preserved. Dietary assessment indicated caloric and protein intake below recommended levels. Anthropometric measurements at the time of dental admission revealed a weight of 10.9 kg and length of 87 cm, both below −2 standard deviations for age, and a reduced mid-upper arm circumference, consistent with moderate acute malnutrition. Laboratory investigations demonstrated mild microcytic hypochromic anemia (hemoglobin 10.9 g/dL) and leukocytosis (total leukocyte count 14,490/mm^3^) with elevated erythrocyte sedimentation rate, suggesting chronic inflammatory status. Liver and renal function tests were within normal limits. Coagulation profile was within acceptable limits for surgical intervention. These findings collectively indicated a compromised systemic and nutritional status, reinforcing the child's high caries risk and need for comprehensive, definitive management.

**Figure 2:**
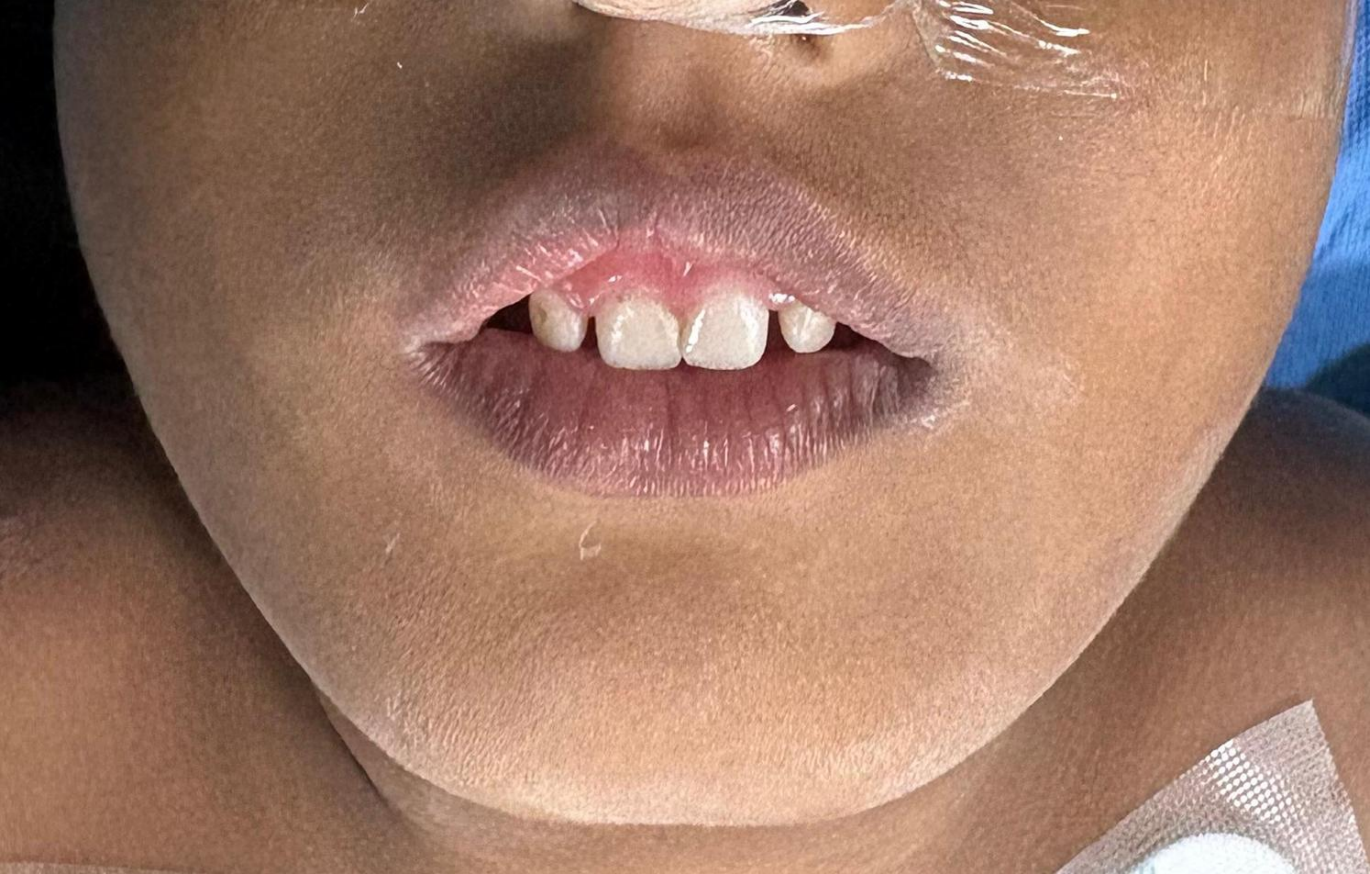
Clinical photograph showing diffuse bilateral enlargement of the mandibular and submandibular regions with normal overlying skin.

On presentation for dental care, the child was irritable, apprehensive, and minimally cooperative for clinical examination. Vital signs were within normal limits. Extraoral examination revealed diffuse bilateral enlargement of the mandibular and submandibular regions with normal overlying skin, absence of local warmth, and no restriction in mandibular movements (Figure 2). Palpation revealed firm bony prominence in the preauricular region consistent residual periosteal thickening. No signs suggestive of acute odontogenic infection were noted.

Intraoral examination demonstrated poor oral hygiene with generalized plaque accumulation. Multiple cavitated dentinal carious lesions were identified. Teeth 64, 74, 75, and 84 exhibited active cavitated lesions involving dentin without clinical signs of pulpal involvement. Tooth 52 presented with a labial carious lesion affecting esthetics and plaque retention. Tooth 54 demonstrated extensive coronal destruction with insufficient remaining tooth structure to support restoration and was diagnosed as nonrestorable. Deep pits and fissures with high caries susceptibility were noted on teeth 55, 65, and 85. No intraoral swelling, sinus tract, or mucosal pathology was observed. Caries experience was assessed using dmft criteria (dmft = 6), and based on clinical findings, systemic status, dietary factors, and developmental limitations, the child was categorized as high caries risk.

Considering the child's young age, developmental delay, extent of treatment required, history of infantile cortical hyperostosis with residual mandibular changes, and anticipated lack of cooperation, comprehensive full mouth rehabilitation under general anesthesia was planned to ensure definitive treatment in a single visit and to minimize cumulative physiological and psychological stress. General anesthesia was specifically indicated due to the combination of extensive treatment need, inability to achieve adequate cooperation, and the need to avoid repeated appointments that could exacerbate systemic stress in a medically compromised child. Preoperative evaluation was conducted in consultation with pediatric and anesthesiology teams to assess systemic stability, airway considerations in view of mandibular enlargement, and perioperative risk. After detailed discussion of risks, benefits, and alternatives, informed written consent was obtained from the parents.

**Figure 3:**
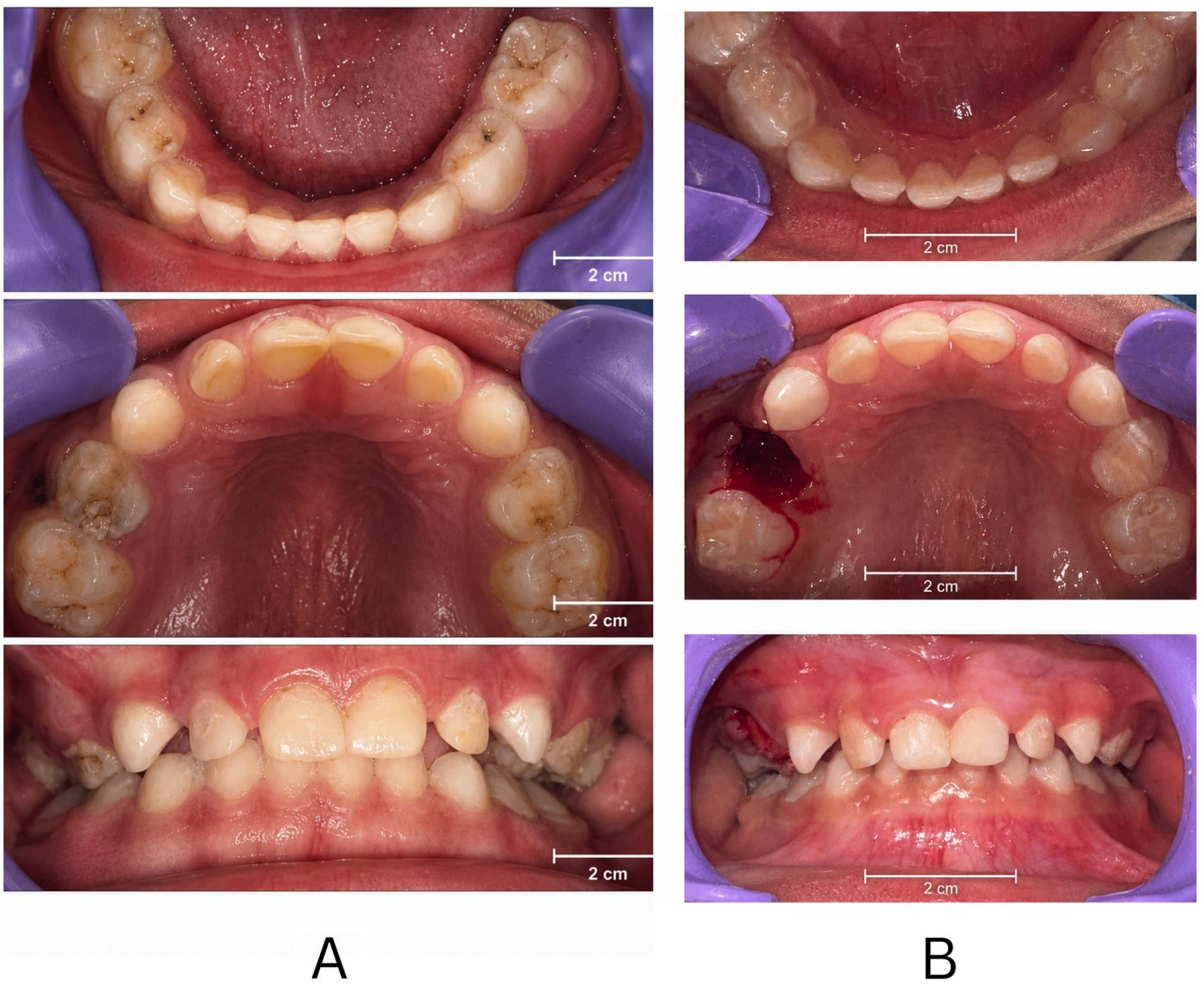
**(A)** Pre-operative intraoral assessment. **(B)** Post-operative intraoral assessment.

Under general anesthesia with standard monitoring, anesthesia was induced using intravenous propofol and fentanyl, followed by maintenance with sevoflurane in oxygen and nitrous oxide. Endotracheal intubation was performed uneventfully after administration of a short-acting neuromuscular relaxant. Continuous monitoring of pulse rate, oxygen saturation, noninvasive blood pressure, and end-tidal carbon dioxide was maintained throughout the procedure. Full mouth oral prophylaxis was performed. Caries excavation was completed, and glass ionomer cement restorations were placed in teeth 64, 74, 75, and 84, selected for their fluoride-releasing properties, chemical adhesion, and suitability in highcaries-risk and moisture-compromised pediatric settings. A composite resin restoration was placed in tooth 52 to restore anterior esthetics and function. Tooth 54 was extracted atraumatically with preservation of surrounding tissues due to inadequate remaining tooth structure and poor long-term prognosis. The extraction socket was irrigated with povidone-iodine and sterile saline, followed by placement of absorbable gelatin sponge to achieve hemostasis. Pit and fissure sealants were applied to teeth 55, 65, and 85 as a preventive measure against future occlusal caries. At the conclusion of the procedure, topical bifluoride varnish was applied to enhance remineralization and reduce recurrence risk. The procedure was completed uneventfully within a single session. Intraoperative and postoperative periods were stable, with no airway compromise, excessive bleeding, or other complications. The child recovered satisfactorily from anesthesia and demonstrated improved oral comfort and feeding tolerance (Figures 3A,B). Postoperative medications included oral paracetamol (15 mg/kg/dose every 6 h as needed for pain for 3 days) and amoxicillin-clavulanate (40 mg/kg/day in divided doses for 5 days) as prophylaxis against postoperative infection following extraction. Parents were instructed regarding medication adherence and monitoring for adverse effects. Intraoperative and postoperative periods were stable. Short-term follow-up at 3 months demonstrated satisfactory restoration integrity, absence of new carious lesions, and improved caregiver-reported oral hygiene practices.

Parents were provided with detailed, individualized oral hygiene instructions tailored to the child's developmental limitations and high caries risk profile. Caregivers were instructed to perform twicedaily supervised brushing using a soft, small-headed toothbrush with 1,000 ppm fluoridated toothpaste in a smear-to-pea-sized quantity appropriate for age. Given the child's limited gross motor coordination, independent brushing was discouraged, and complete caregiver-assisted plaque removal was emphasized, particularly along the gingival margins and occlusal surfaces of posterior teeth. Demonstration of the modified knee-to-knee positioning technique was provided to facilitate safe and effective home brushing. Caregivers were also advised to wipe the teeth with gauze after meals when brushing was not immediately feasible, especially during episodes of irritability or systemic flare-ups.

Dietary counseling was conducted in coordination with pediatric recommendations to address both nutritional rehabilitation and caries prevention. Parents were educated on increasing protein-rich and micronutrient-dense foods to support growth and hematologic recovery while limiting the frequency of fermentable carbohydrate exposure. Emphasis was placed on structured meal timing, elimination of nocturnal feeding practices, avoidance of sweetened beverages, and encouraging water intake after meals. The role of adequate calcium and vitamin D intake in supporting skeletal health was reinforced in view of the underlying bone disorder.

Specific anticipatory guidance was provided regarding recognition of recurrent mandibular inflammatory episodes. Parents were instructed to report sudden increases in facial swelling, pain, drooling, feeding refusal, or unexplained irritability. The importance of differentiating between recurrence of Caffey-related periostitis and possible odontogenic infection was explained, and they were advised to seek prompt dental and pediatric evaluation if localized tenderness, purulence, or intraoral swelling was noted. In addition, the child was referred by the pediatric team to a child psychologist/psychiatrist for behavioral evaluation and parental counseling to support coping with recurrent painful episodes and associated distress.

A structured recall protocol was established with follow-up visits scheduled at three-month intervals due to the child's high caries risk status. Each recall visit was planned to include professional fluoride varnish application, reinforcement of oral hygiene techniques, dietary reassessment, and evaluation of restoration integrity. Additionally, longitudinal monitoring of eruption sequence, occlusal development, and mandibular growth symmetry was incorporated into the follow-up plan to detect any deviations potentially related to prior cortical hyperostosis. Coordination with the pediatric team was maintained to ensure alignment between dental surveillance and systemic growth monitoring.

**Figure 4:**
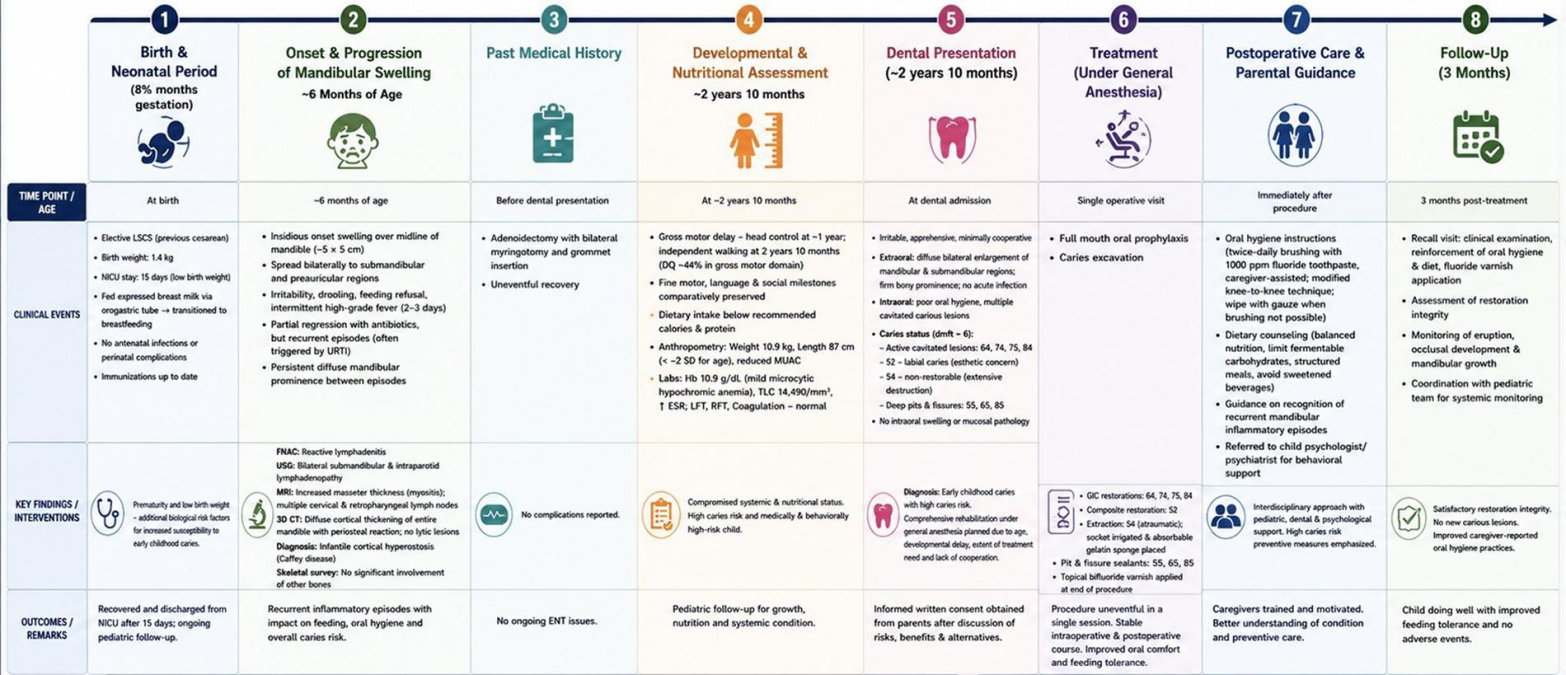
Clinical timeline summarizing the chronological sequence of events, including symptom onset, diagnostic evaluation, relevant medical history, dental findings, therapeutic interventions, and follow-up outcomes.

As reported by the caregiver, the child demonstrated improved feeding tolerance and reduced irritability following treatment. Oral hygiene practices were easier to perform after structured instruction, and the caregiver expressed satisfaction with the overall outcome. No concerns or adverse events were reported during follow-up. In accordance with the CARE (CAse REport) guidelines, a structured clinical timeline has been incorporated to summarize the chronological sequence of events, including symptom onset, diagnostic evaluation, relevant medical history, dental findings, therapeutic interventions, and follow-up outcomes (Figure 4).

## 3 Discussion

Infantile cortical hyperostosis (Caffey disease) is an uncommon, self-limiting inflammatory disorder characterized by episodic subperiosteal bone formation, most frequently involving the mandible, clavicle, and long bones (1, 2). Although spontaneous resolution is typical, residual mandibular changes and functional sequelae during early infancy may occur at a critical period of craniofacial growth and primary dentition development, potentially influencing oral function and hygiene practices; however, direct effects on dental tissues remain inadequately documented in current literature (7).

The present case illustrates a clinically plausible, multifactorial risk pathway in which sequelae of prior inflammatory episodes, nutritional compromise, developmental delay, and behavioral limitations coexisted with early childhood caries (ECC). Mandibular cortical thickening with periosteal reaction, as demonstrated radiographically, was associated with episodic pain and feeding difficulty in this patient; however, its direct contribution to ECC progression cannot be established and is interpreted as indirect and context-dependent. In early childhood, even transient reductions in effective oral hygiene can promote demineralization due to the relatively thin enamel of primary teeth (8). Thus, the observed caries burden is more appropriately understood as the cumulative effect of multiple risk modifiers rather than a direct manifestation of the underlying skeletal disorder.

The interaction between malnutrition and ECC in this case reflects a well-recognized but complex association. Anthropometric parameters below −2 standard deviations and laboratory evidence of anemia indicated systemic vulnerability. Malnutrition has been associated with impaired host response and altered salivary function, potentially increasing caries susceptibility (9). Conversely, untreated ECC may exacerbate feeding discomfort and reduce dietary intake (9). While this bidirectional relationship is supported in broader pediatric literature, its specific interaction with Caffey disease remains insufficiently studied and should be interpreted cautiously.

Developmental delay introduced additional behavioral complexity. Limited gross motor coordination necessitated full caregiver-dependent plaque control, while behavioral immaturity precluded predictable cooperation for conventional chairside care. Current pediatric dental guidelines endorse general anesthesia (GA) when extensive treatment is required in young or medically compromised children in whom behavior guidance techniques are unlikely to achieve safe and efficient outcomes (6). In this case, GA was selected based on individualized assessment of disease extent, cooperation level, and systemic considerations, rather than as a disease-specific requirement.

Airway considerations merit particular attention in patients with mandibular hyperostosis (10, 11). Although no airway difficulty was encountered in this case, cortical thickening and soft tissue changes may theoretically influence airway assessment and mask adaptation. Therefore, preoperative interdisciplinary evaluation was undertaken as a precautionary measure rather than in response to documented airway compromise. This distinction is important to avoid overinterpretation of anesthetic risk.

Restorative material selection was guided by high caries risk status, anticipated compliance challenges, and the need for fluoride-mediated secondary prevention. Glass ionomer cement (GIC) was preferred for posterior restorations because of its chemical adhesion, fluoride release, and tolerance to suboptimal moisture control—advantages particularly relevant in toddlers with limited cooperation (12). Composite resin was reserved for anterior esthetic rehabilitation to improve surface smoothness and reduce plaque retention. Alternative options such as stainless steel crowns were considered; however, given the extent and pattern of lesions, along with the clinical objective of minimally invasive rehabilitation, direct restorations were deemed appropriate in this case. Extraction of the non-restorable molar was performed atraumatically. Notably, despite radiographic evidence of cortical thickening, no increased resistance to extraction was encountered. This observation contrasts with theoretical concerns regarding bone density and suggests that dentoalveolar surgical procedures may not be significantly compromised in all cases of Caffey disease. However, this finding is based on a single observation and requires further validation.

Preventive reinforcement constituted a core component of management. Sealant placement and topical fluoride varnish were implemented intraoperatively; however, long-term disease control depends primarily on caregiver-mediated behavioral modification. Recommendations were therefore individualized and aligned with the child's developmental limitations and systemic condition, rather than based on disease-specific protocols, which are currently lacking. Structured counseling targeted reduction in fermentable carbohydrate frequency, elimination of nocturnal feeding, and supervised twice-daily brushing with fluoridated toothpaste. Given the child's systemic condition, anticipatory guidance also included differentiation between inflammatory mandibular recurrence and odontogenic infection, as both may present with facial swelling but require distinct management pathways (13).

From a craniofacial perspective, early mandibular hyperostosis may influence growth patterns; however, no immediate occlusal or eruption abnormalities were observed in this patient (7). Long-term monitoring is therefore essential to identify any delayed effects. At present, evidence linking Caffey disease to altered occlusal development remains limited. Periodic evaluation of eruption sequence, arch symmetry, and jaw growth is recommended until skeletal maturation, particularly in children with early-life inflammatory bone disorders (13).

The literature on dental management in Caffey disease remains limited, with most reports focusing on radiologic and pediatric diagnostic considerations rather than oral rehabilitation strategies (2, 7, 11, 13). This case adds to the limited body of evidence by describing practical considerations in dental rehabilitation; however, it does not establish causative relationships or generalizable treatment protocols. Importantly, increased clinical awareness of infantile cortical hyperostosis is essential because unfamiliarity with this rare condition may lead to unnecessary invasive investigations, misdiagnosis, and empirical therapeutic interventions during episodes of facial swelling or irritability. Recognition of its characteristic clinical and radiographic features can support timely diagnosis and help avoid avoidable procedures and treatments. Furthermore, this case highlights that systemic inflammatory bone disorders may indirectly amplify ECC risk through behavioral, nutritional, and functional pathways rather than through direct dental structural abnormalities.

Nevertheless, certain limitations must be acknowledged. This is a single case report with short-term follow-up, limiting the ability to assess long-term outcomes, recurrence of caries, or craniofacial growth changes. Genetic confirmation of COL1A1 mutation was not available. Additionally, although dmft was recorded, more comprehensive longitudinal caries assessment and radiographic follow-up would strengthen clinical interpretation. Future studies and case series are needed to develop evidencebased guidance for dental management in rare pediatric skeletal disorders.

## 4 Conclusion

This case describes the dental management of a child with sequelae of infantile cortical hyperostosis presenting with severe early childhood caries in the context of developmental delay and malnutrition. The findings highlight the need for individualized, multidisciplinary planning when managing oral health in children with rare systemic conditions. In this instance, comprehensive rehabilitation under general anesthesia enabled definitive management in a single visit; however, this approach was based on patient-specific factors and should not be generalized. The case also underscores the importance of integrating medical, nutritional, and behavioral considerations into caries risk assessment and treatment planning. Given the inherent limitations of a single case report and short-term follow-up, broader clinical recommendations cannot be drawn. Further documentation of similar cases is required to inform evidence-based dental management strategies in this population.

## Data Availability

The original contributions presented in the study are included in the article/Supplementary Material, further inquiries can be directed to the corresponding authors.
